# Bicuspidization of the Unicuspid Aortic Valve: Neocommissure Reconstruction Preserving the Native Free Margin

**DOI:** 10.1016/j.atssr.2026.01.002

**Published:** 2026-01-20

**Authors:** Ryo Kawabata, Kotaro Tsunemi, Takanori Oka, Yutaka Okita

**Affiliations:** Department of Cardiovascular Surgery, Cardio-Aortic Center, Takatsuki General Hospital, Osaka, Japan

## Abstract

This report presents a bicuspidization technique for unicuspid aortic valve repair. To enhance long-term durability, the native free margin was preserved, and a portion was used to construct a new commissure, thereby minimizing the use of the patch in regions subjected to high mechanical stress. At 6.5 years postoperatively, the valve function remains excellent.

Bicuspidization techniques for repairing unicuspid aortic valves have been reported, many of which involve modifying cusp geometry and reconstructing new commissures or cusps using pericardial patches.^1^^,^^2^

In the context of aortic valve repair durability, considerable attention has been given to the type of patch material and treatment method.^3^ However, less attention has been paid to the specific anatomical location of patch suturing.

Fluid-structure interaction analysis by Marom and associates^4^ revealed that shear stress is highest at the coaptation edge and commissural regions, suggesting that these areas are critical for valve durability.

In this report, we describe a bicuspidization technique in which the native free margin was preserved, and a part of it was incorporated into a new commissure. This allowed the use of the patch to be limited to the cusp belly, with the aim of improving long-term durability.

The patient was a 35-year-old man who had been diagnosed with aortic regurgitation (AR) at age 17 years. Serial echocardiography revealed a progressive worsening of the AR. The body surface area was 2.08 m^2^. Transesophageal echocardiography revealed annuloaortic ectasia with severe AR. The aortoventricular junction, sinus of Valsalva, and sinotubular junction diameters were 32, 43, and 36 mm, respectively. The left ventricular end-diastolic diameters and end-systolic diameters were 63 mm and 41 mm, respectively, and the left ventricular ejection fraction was 56%. The aortic valve comprised 1 normal commissure with 2 calcified raphes in the anteroposterior direction (Sievers type II) (Figures 1A, 1B).

**Figure 1:**
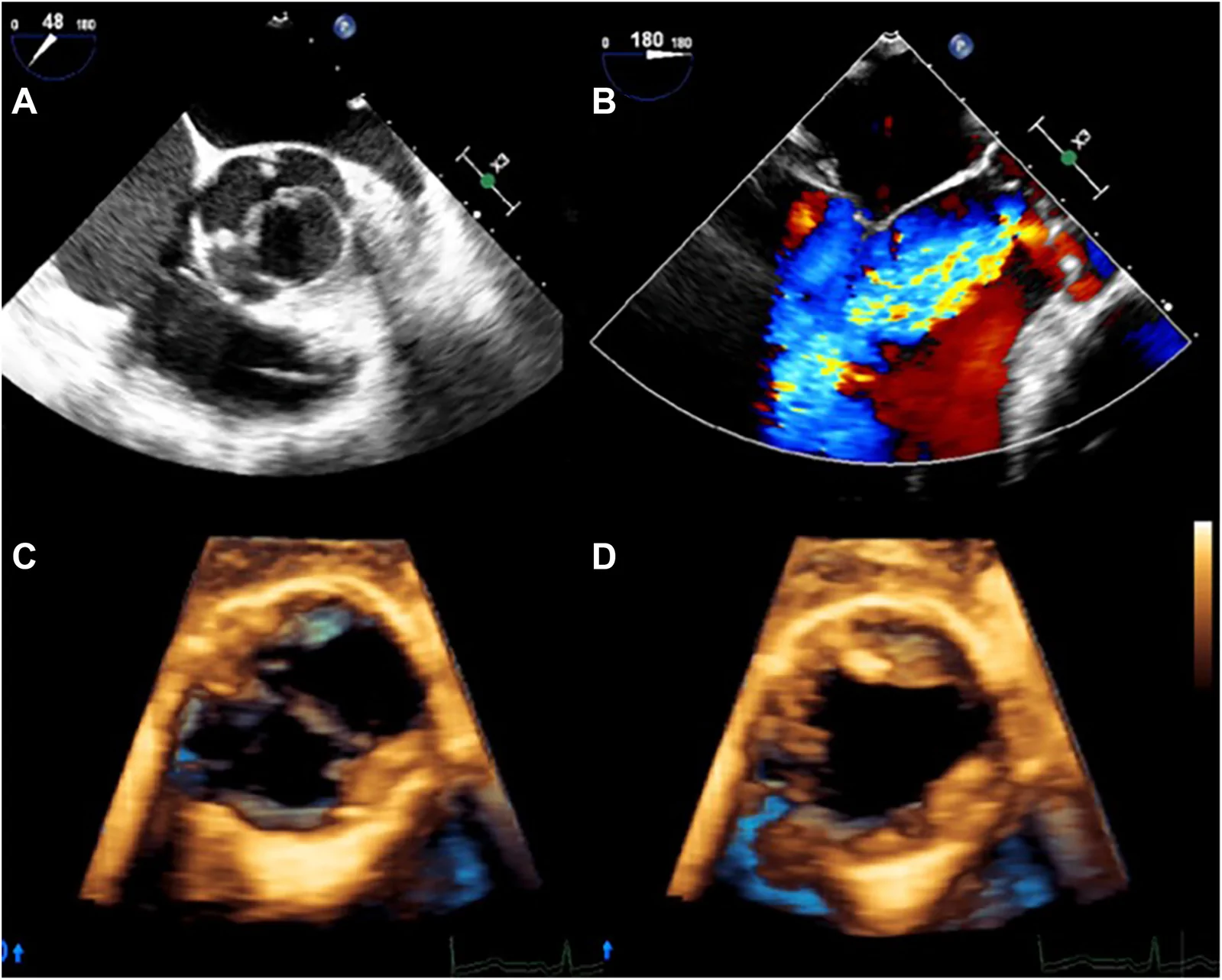
Preoperative transesophageal echocardiography, (A) short-axis and (B) long-axis views. Postoperative transesophageal echocardiography revealed satisfactory (C) valve opening and (D) closure.

## Technique

**Figure 2:**
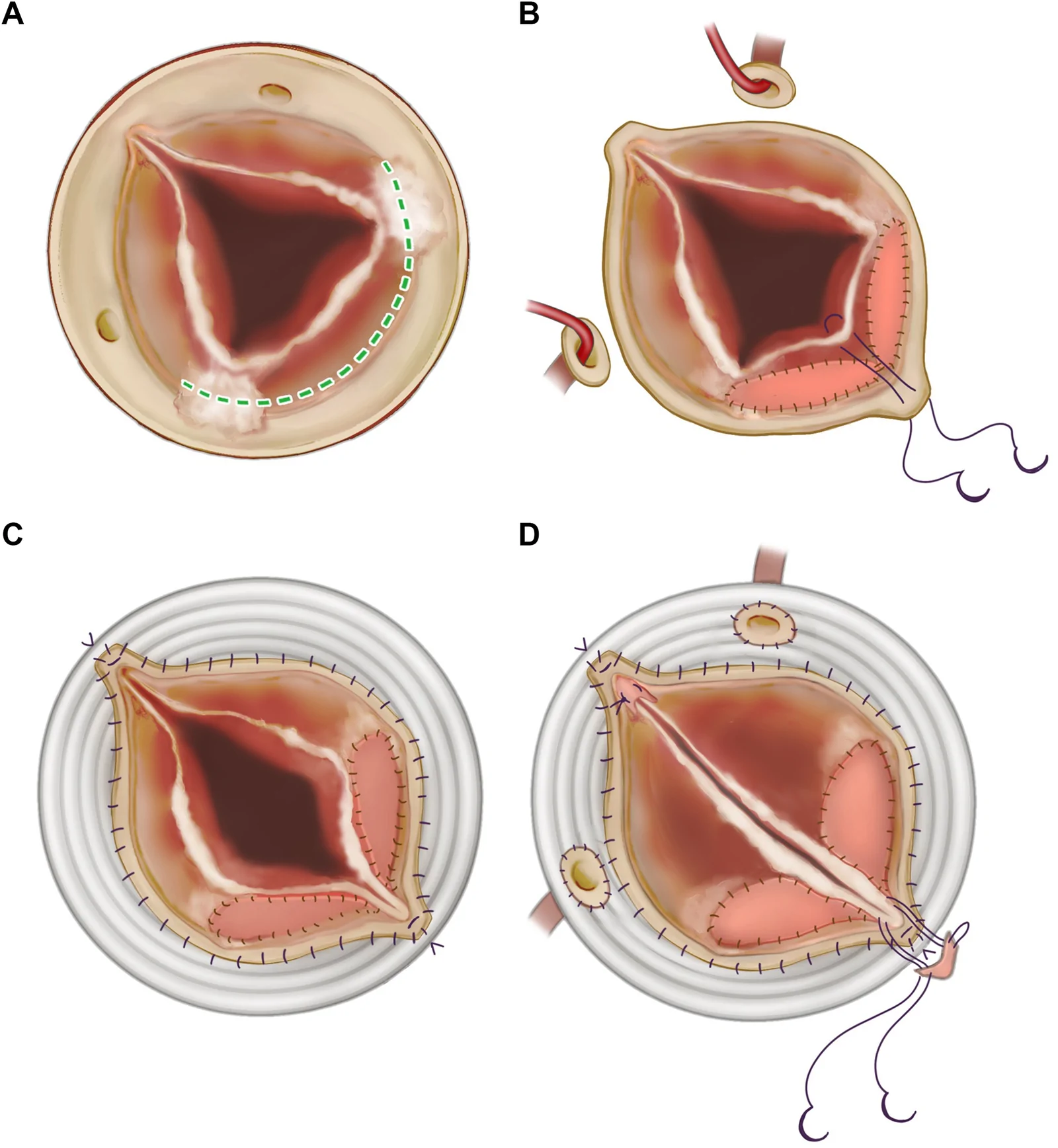
(A) 120° circumferential incision along the aortic annulus at the cusp insertion. (B) Suturing of the treated autologous pericardial patches for the cusp belly and the new commissure was created at a position symmetrical to the native commissure with a portion of the free margin and anchored to the aortic wall. (C) The aortic root was reconstructed using a reimplantation technique with a 28-mm Dacron Valsalva graft (Terumo). Both commissures were made at 180° to the graft sinotubular junction. (D) Two commissures were tightened with pericardial buttress sutures to approximate the gap. And the coronary buttons were anastomosed to the graft, completing the procedure.

Median sternotomy and cardiopulmonary bypass were performed on the patient. After aortic transection, the unicuspid valve was recognized as a normal commissure located on the left and 2 calcified raphes in the anterior and posterior-right positions. The height of the 2 raphes was lower than that of the normal commissure. The geometric height of the cusp was 24 mm. The raphe tissues were then shaved and decalcified. Subsequently, a 120° circumferential incision along the aortic annulus at the cusp insertion was made to the anterior right (Figure 2A). Two rectangular autologous pericardial patches (1.0 cm × 1.5 cm), which had been treated with 0.6% glutaraldehyde solution for 10 minutes, were sutured to the cusp belly for cusp enlargement using 5-0 polypropylene sutures. The new commissure was created at a position opposite to the native commissure with a portion of the free margin and anchored to the aortic wall (Figure 2B).

The aortic root was reconstructed using a reimplantation technique with a 28-mm Dacron Valsalva graft (Valsalva Ante-flo; Terumo).

Both commissures were positioned 180° apart along the graft sinotubular junction (Figure 2C). The effective height was 10 mm on both sides. After shaving the thickened free margin, the two commissures were tightened with pericardial buttress sutures to approximate the gap. Finally, the coronary buttons were anastomosed to the graft, completing the procedure (Figure 2D). Postoperative transesophageal echocardiography revealed satisfactory valve opening and closure (Figures 1C, 1D) (Supplemental Video).

**Figure 3:**
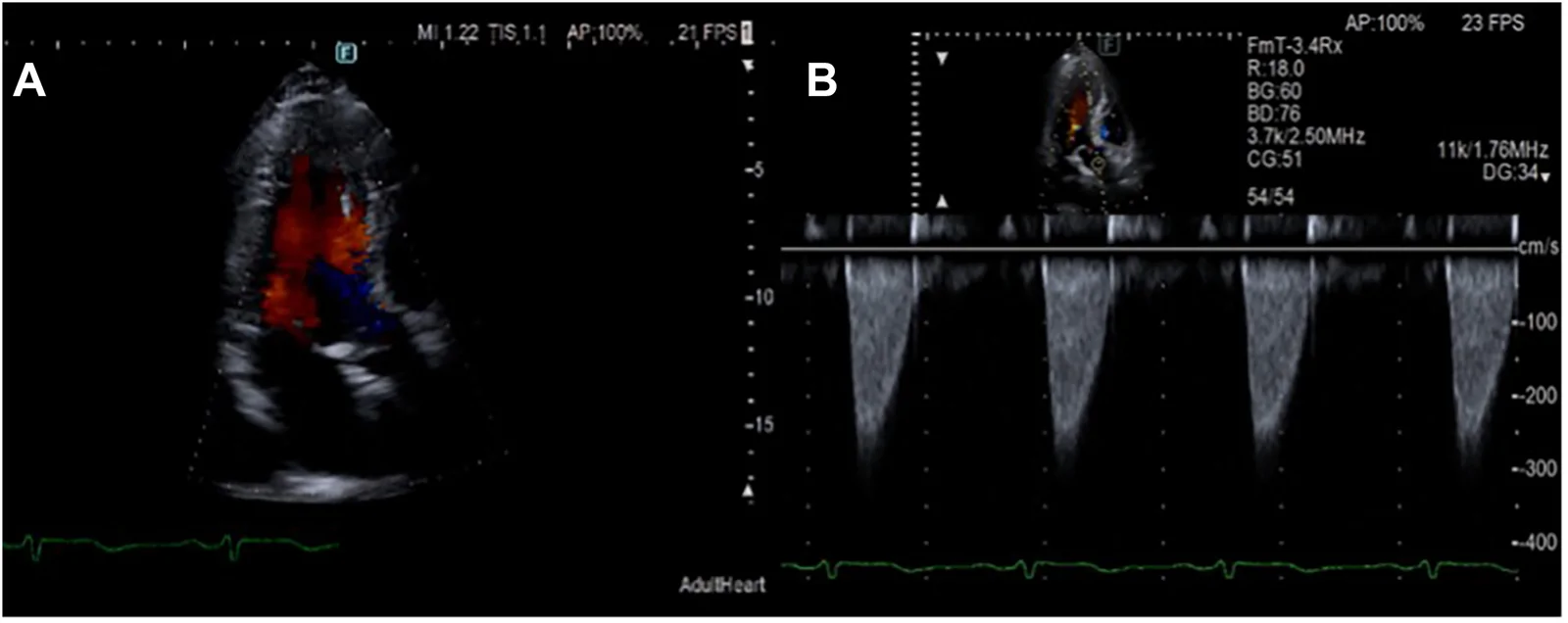
Transthoracic echocardiography at 6.5 years postoperatively. (A) Apical long-axis view showing trace aortic regurgitation. (B) The mean pressure gradient was 15 mm Hg, indicating no evidence of stenosis.

Transthoracic echocardiography at discharge showed trace AR, with a mean pressure gradient of 16 mm Hg and an effective orifice area index of 0.83 cm^2^/m^2^. At 6.5-year follow-up, transthoracic echocardiography remained stable with trace AR (mean pressure gradient, 15 mm Hg; effective orifice area index, 0.77 cm^2^/m^2^) (Figures 3A, 3B).

## Comment

The primary concept of the proposed technique lies in the strategic placement of pericardial patches to enhance long-term durability. In conventional bicuspidization techniques, pericardial patches are frequently used at the commissures and along the free margin, regions that are known to experience high shear stress within the aortic valve.^4^ Fluid-structure interaction analysis by Marom and colleagues^4^ demonstrated that the commissures and coaptation zone are the areas of greatest shear stress concentration, indicating that the use of patches in these zones may predispose patients to degeneration and suture dehiscence.

In the current technique, pericardial patches were confined to the cusp belly, whereas the coaptation zone or the commissure, where shear stress is maximal, was entirely reconstructed from native leaflet tissue. This approach may help reduce the risk of material fatigue and structural failure. Schäfers and colleagues^5^ reported favorable short- to mid-term outcomes with bicuspidization using decellularized pericardial patches, suggesting that improvements in patch materials may contribute to improved durability. In contrast, our approach emphasizes the location rather than the type of patch material used and has demonstrated excellent functional midterm outcomes.

Regarding surgical alternatives, the Ross procedure was not selected in the present case because of the considerably dilated aortic annulus, which is associated with a higher risk of autograft dilatation. In contrast, Gofus and coworkers^6^ suggested that the Ross procedure may offer superior mid-term durability compared with bicuspidization, highlighting the importance of careful patient selection for each technique.

A limitation of the surgical technique we have presented is the requirement for an adequate length of the free margin. Furthermore, the true value of this procedure will be determined only after accumulating a larger number of cases and obtaining long-term outcome data beyond 10 years.
